# A Liquid State Perspective on Dynamics of Chromatin Compartments

**DOI:** 10.3389/fmolb.2021.781981

**Published:** 2022-01-13

**Authors:** Rabia Laghmach, Michele Di Pierro, Davit Potoyan

**Affiliations:** ^1^ Department of Chemistry, Iowa State University, Ames, IA, United States; ^2^ Department of Physics, Northeastern University, Boston, MA, United States

**Keywords:** chromatin, liquid-liquid phase separation, heterochromatin, euchromatin, nuclear organization, lamin, imaging, mesoscale

## Abstract

The interior of the eukaryotic cell nucleus has a crowded and heterogeneous environment packed with chromatin polymers, regulatory proteins, and RNA molecules. Chromatin polymer, assisted by epigenetic modifications, protein and RNA binders, forms multi-scale compartments which help regulate genes in response to cellular signals. Furthermore, chromatin compartments are dynamic and tend to evolve in size and composition in ways that are not fully understood. The latest super-resolution imaging experiments have revealed a much more dynamic and stochastic nature of chromatin compartments than was appreciated before. An emerging mechanism explaining chromatin compartmentalization dynamics is the phase separation of protein and nucleic acids into membraneless liquid condensates. Consequently, concepts and ideas from soft matter and polymer systems have been rapidly entering the lexicon of cell biology. In this respect, the role of computational models is crucial for establishing a rigorous and quantitative foundation for the new concepts and disentangling the complex interplay of forces that contribute to the emergent patterns of chromatin dynamics and organization. Several multi-scale models have emerged to address various aspects of chromatin dynamics, ranging from equilibrium polymer simulations, hybrid non-equilibrium simulations coupling protein binding and chromatin folding, and mesoscopic field-theoretic models. Here, we review these emerging theoretical paradigms and computational models with a particular focus on chromatin’s phase separation and liquid-like properties as a basis for nuclear organization and dynamics.

## 1 Introduction

The eukaryotic nucleus is a membrane bound-organelle with a crowded, heterogeneous, and dynamically changing biomolecular composition. A large fraction of the nucleus is occupied by chromatin, a tight association of genomic DNA with histone proteins. The 1D sequence of chromatin polymer is decorated with epigenetic marks, which add an extra layer of information on the top of the DNA sequence. The 3D conformations of chromatin in the nucleus evolve over cellular life because of the passive diffusion and binding of biomolecules in the nucleoplasm and the active processes acting on conformations and epigenetic states of chromatin. Despite the molecular stochasticity and dynamism present in the nucleus, the chromatin organization and dynamics are not random but highly correlated with transcriptional activities and phenotypic states of cells.

A fundamental question in genome biophysics is to understand the link between three layers of information encoded by chromatin and its environment; 1D epigenetic patterning, 3D architectures, and transcriptional processes. Historically, different experimental and computational techniques have been invented for interrogating different scales of chromatin, ranging from single nucleosomal units to folding of chromosomes and mesoscale chromatin organization in the nucleus. Recent advances in single-cell imaging techniques, computational modeling, and machine learning methods have brought experiment and theoretical approaches much closer, calling for more integrative analysis and interpretation of chromatin behavior by bridging spatial and temporal scales.

A testament to this is the founding of the 4D nucleome initiative Dekker et al. (2017) which aims to map 3D architectural models across time and space, thereby linking gene expression machinery and other biological functions to detailed chromatin conformational dynamics. Over the last decade, a great deal has been learned about the static 3D organization of chromatin thanks to the chromosome conformation capture techniques, especially the Hi-C Lieberman-Aiden et al. (2009a); van Berkum et al. (2010). There are many excellent reviews summarizing the current state of knowledge on structural aspects of 3D chromatin folding Jerkovic and Cavalli (2021); Ghosh and Meyer (2021); Grigoryev and Schubert (2019); Cremer et al. (2020); Itoh et al. (2021a); therefore, we only briefly list the significant findings that have direct implications for discussing chromatin dynamics.

**FIGURE 1:**
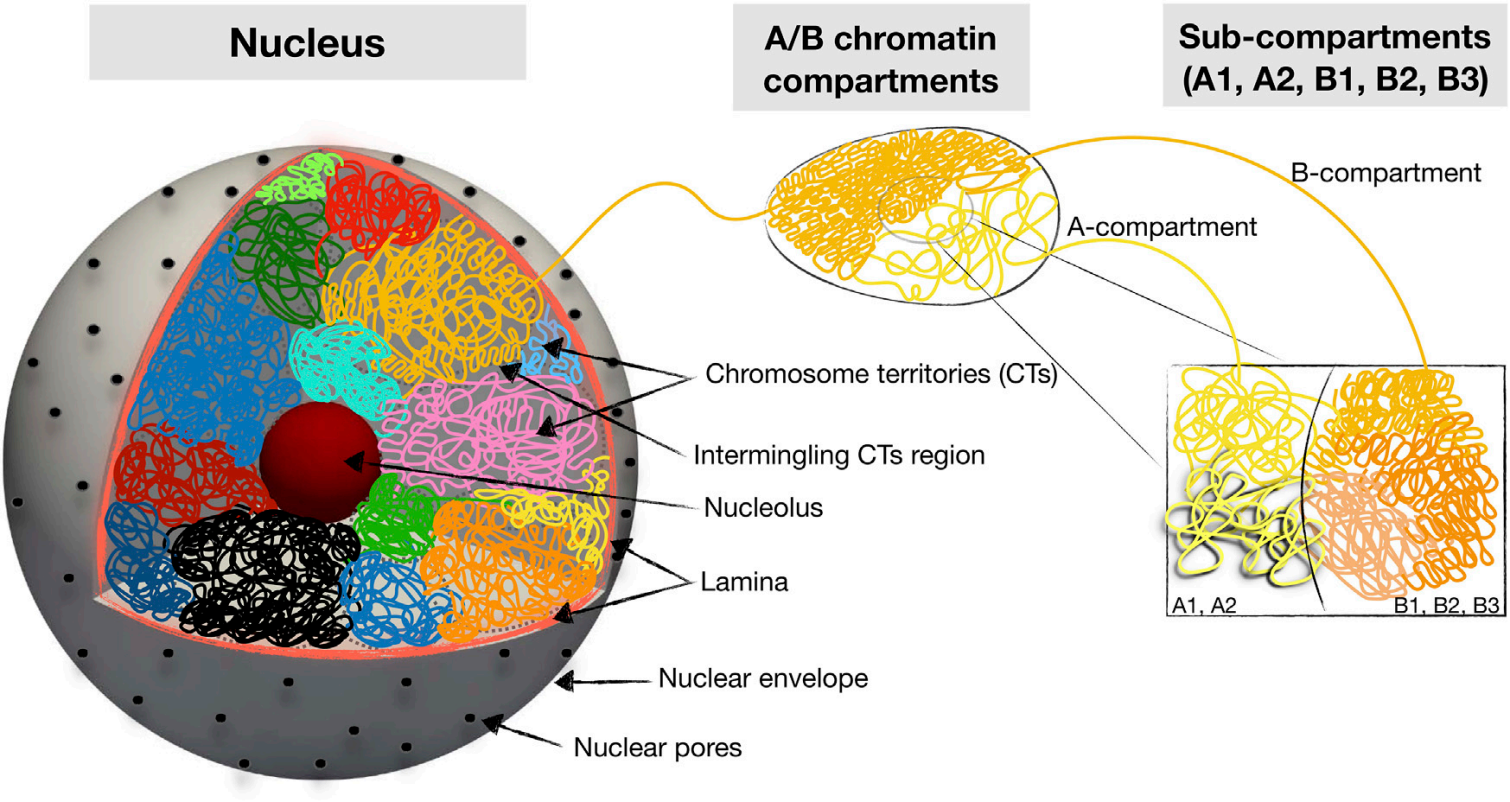
Schematic summary of hierarchical 3D folding of chromatin into compartments and domains. Shown are various keywords relevant for describing nuclear chromatin architecture along with length scales relevant for modeling and imaging studies.

Chromatin is self-organized in a hierarchical manner (Figure 1). At the nuclear scale, the chromosomes are organized into individual territories (CTs), which have nonrandom positioning relative to the nuclear center Cremer and Cremer (2001). Within the chromosomal territories, chromatin is partitioned into two spatial compartments, which, on the basis of epigenetic tracks and Hi-C maps, are quantitatively classified into A and B types. The compartmentalized A/B chromatin states strongly overlap with euchromatin and heterochromatin compartments, which are physically distinct states of chromatin differing in density, transcription activity, and histone modifications Lieberman-Aiden et al. (2009b); Dekker et al. (2013); Rao et al. (2014). Specifically, the euchromatin compartment is defined as the less dense genomic regions that are enriched by transcriptionally active genes and histone marks, while the heterochromatin compartment is defined as the dense genomic regions associated with transcriptionally inactive genes and repressive histone modifications. The spatial partition of the A and B compartments is cell type-specific with a strong dependence on the cell cycle and line Dixon et al. (2015); Su et al. (2020); Feodorova et al. (2020). At the intermediate scale, these 2 A/B compartments themselves have substructures referred to as topologically associated domains (TADs), which in turn are organized into smaller nano-domains and loops Dekker et al. (2013); Rao et al. (2014).

To date, the majority of Hi-C data has been collected at the cell population level. The recently single-cell Hi-C and super-resolution imaging reveal a much more stochastic and dynamical behavior of chromatin throughout cell cycle and differentiation. This picture of chromatin is consistent with active multiphase liquid condensate Shaban et al. (2018); Shaban and Seeber (2020); Barth et al. (2020); Miron et al. (2020). Further support for multiphase condensate ideas comes from experiments on proteins and nucleic acids liquid-liquid phase separation (LLPS) Brangwynne et al. (2009); Brangwynne et al., 2011; Nott et al. (2015); Molliex et al. (2015); Pak et al. (2016). These findings imply that the liquid behavior strongly impacts chromatin dynamics and multivalent interactions with nuclear proteins and RNA peng and Weber (2019); Maeshima et al. (2020); Sanulli and Narlikar (2020); Choi et al. (2020); Smith et al. (2020). The notion of liquid chromatin states Maeshima et al. (2020) is further supported by the observations of the viscoelastic response of chromosomal loci Zidovska et al. (2013); Lucas et al. (2014); Di Pierro et al. (2018); Strickfaden et al. (2020), coherent motions of chromatin domains Zidovska et al. (2013); Saintillan et al. (2018), the coalescence and Ostwald ripening of chromatin droplets Caragine et al. (2018); Lee et al. (2021), the existence of epigenetic zonations and chains of interlinked ∼200,−,300nm wide chromatin domains reminiscent of polymer melts Miron et al. (2020). The dynamical liquid-like behavior of chromatin is usually interpreted in terms of the microphase separation mechanism. The epigenetically decorated chromosomes consisting of effective A/B immiscible types act as long copolymers that microphase separate, forming several stable droplets/clusters. An interesting contribution from Gibson et al. Gibson et al. (2019) has revealed that liquid chromatin droplets fuse rapidly, but the rate of content mixing is very slow, as a result of which the degree of phase separation is low. By considering the chromatin as a block copolymer that can fold in restricted space which is furthermore juxtaposed by the nuclear constraints, one naturally predicts the formation of smaller clusters relative to a pure copolymeric system [Gibson et al. (2019); Hildebrand and Dekker (2020)]. The presence of non-equilibrium, motorized ATP-driven processes is also shown to modulate chromatin dynamics which manifests in the form of ATP-dependent flows, driven fluctuations, and anomalous diffusion coefficients of chromatin loci Chu et al. (2017); Saintillan et al. (2018). Thanks to these and other developments, a liquid chromatin perspective is increasingly gaining a foothold in the field of chromatin biophysics. From this perspective, the chromatin at mesoscopic scale is seen as a complex fluid material that can phase separate and form membraneless compartments that can grow, fuse and dissolve in response to environmental triggers. Many bio-molecular components of the nucleus, including chromatin, transcription factors, and nuclear bodies, have already been observed to undergo phase separation. These developments have propelled liquid-liquid phase separation as an important mechanism underlying nuclear organization and dynamics.

To dissect the complexity of nuclear chromatin organization and dynamics requires an inter-disciplinary approach combining experimental techniques to theoretical, mathematical, and physical modeling. Various computational models have been developed for addressing questions relevant to a particular chromatin scale. The models range from explicit all-atom simulations of nucleosomes to coarse-grained polymer models of chromosomes and to continuous mechanical models of the cell’s nucleus. Herein we will focus specifically on the models that embrace the liquid dynamical perspective of chromatin and make concrete predictions about the observed phenomena. These models’ continuous development and refinement will be essential for understanding the roles and impact of phase separation on emergent chromatin organization and dynamics.

### 2 Polymer Models of Phase Separation and Dynamics of Chromatin Compartments

Fluorescence *in situ* hybridization (FISH) techniques have established that interphase chromosomes occupy distinct nuclear regions known as chromosome territories within which chromosomes can be seen as polymers with nonrandom 3D folds Cremer and Cremer (2001). Therefore it is natural to appeal to the ideas of polymer physics for interpreting the data on chromatin folding and dynamics. Furthermore, computer simulations with polymeric models enable one to make concrete predictions on experimentally accessible quantities: frequency of contacts between two chromosomal loci, diffusion coefficients, spatial and temporal correlation functions, the impact of looping and chain entanglement on 3D architectures, etc. Before delving into the predictions made by various polymer models and simulations, it is instructive to review the basic assumptions that underlie polymer simulations of chromatin.

The two most common polymer models used for folding chromosomes are derived either *via* a forward or inverse approaches Tiana and Giorgetti (2019); Fiorillo et al. (2020); Brackey et al. (2020); Lin et al. (2021); Di Stefano et al. (2021a); Yildirim et al. (2021); Chiang et al. (2022). In the forward approach (also known as mechanistic approach), one first designs, an energy function that describes chromosomal loci interactions mainly based on accumulated evidence from experiments while relying on the physical intuition from polymer physics. In the forward approach, the input parameters of the designed potential energy function used in the polymer models are varied for testing a specific hypothesis in order to reproduce experimental data or for generating new ideas which guide the experiments. The flexibility and freedom of the forward approach allow navigating the parameter space and discovering insights that are not readily seen in the available data.

The forward or mechanistic approach has been widely used to investigate various biological questions related to the physical properties of DNA; the microphase separation of DNA into the nucleus, formation of protein-mediated chromatin loops, and genome organization Barbieri et al. (2012); Nuebler et al. (2018); Tiana and Giorgetti (2019); Brackey et al. (2020). In the mechanistic approach, most models used for simulating the whole-genome organization derived from the polymer model in which the usual potential energy function is completed by additional terms for chromatin constraints to describe the inter and intrachromosomal interactions and whose input parameters are derived from the experimental Hi-C maps Barbieri et al. (2012); Nuebler et al. (2018); Tiana and Giorgetti (2019); Brackey et al. (2020).

**FIGURE 2:**
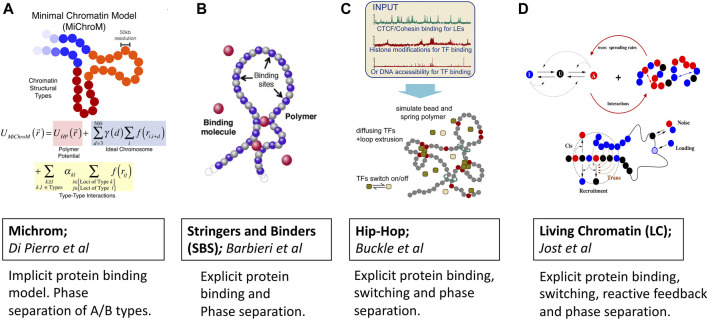
Predictive polymer models of 3D chromatin folding based on protein binding, loop extrusion and phase separation ideas. Images are adopted from original papers with copyright agreement. From left to right; **(A)** Michrom Di Pierro et al. (2018), **(B)** Stringers and Binders Barbieri et al. (2012), **(C)** Hip-Hop Buckle et al. (2018) and **(D)** Living Chromatin Jost and Vaillant (2018).

The inverse approach starts once again from an energy function motivated by a combination of polymer physics and experimental information. The parameters of the model in the inverse approach, however, are trained on a combination of Hi-C, 1D Chip-seq/RNA-seq and/or fluorescence *in situ* hybridization (FISH) data once and then used for making predictions on a new batch of data such as different cell lines or cell cycle stages. The accumulation of high precision Hi-C and FISH data has greatly expanded the reach of polymer models, which now play a crucial role in visualizing chromosome folding and dynamics Di Pierro et al. (2017); Tiana and Giorgetti (2019); Lin et al. (2021) (Figure 2).

A fundamental question regarding the polymeric models which are trained against reproducing contact maps is whether they will be able to recapitulate the dynamic features of chromosomes? The dynamical information contained in FISH and other imaging approaches could, in principle, be used to complement and enhance information in Hi-C. However, the relationship between Hi-C and FISH is far from trivial to reconcile. For instance, Fudenberg et al. Fudenberg and Imakaev (2017) find that transforming ensemble average contact frequencies into spatial distances generates inconsistent models of chromosomal organization. This inconsistency stems from a mixture of conformational sub-populations of chromosomes, each characterized by its own statistics. This global heterogeneity is an intrinsic characteristic of all chromosomes and is the primary reason for the discrepancy between contact frequency and distance distributions obtained by Hi-C and FISH. Therefore, it is clear that for a deeper understanding of chromatin conformations and dynamics, one must at least be able to reconcile and integrate structural information from conformational capture techniques with dynamical information from imaging experiments. To this end, Shi et al. Shi and Thirumalai (2019) have deployed Generalized Rouse Chromosome Model for reconciling the Hi-C and FISH data. Recently, Onami’s group has proposed a simulation tool using a streamlined polymer network model called Phi-C to bridge the information gap between Hi-C and imaging experiments Shinkai et al. (2020).

The growing amount of data provided by Hi-C and imaging technologies coupled with the increasing resolution has bolstered the development of computational models that are becoming increasingly more quantitative in their predictions of complex structural aspects and underlying mechanisms behind chromatin folding and dynamics. Initially, models were as homo-polymeric chains, but over time as the realization of the importance of protein binding, bridging, and phase separation emerged, hetero-polymer models became more common. A beautiful illustration of a predictive reach of co-polymeric models is provided by a recent study by Falk et al. Falk et al. (2019) which parametrized A/B copolymer model of chromosomes against the Hi-C maps of rods cell and have shown that a judicious interplay of A-A and B-B self-interactions, A-B cross interactions and the strength of the heterochromatin attachment to the nuclear envelope dictate global nature of chromatin compartmentalization underlying conventional and inverted nuclear architectures. In a recent study of nucleoli formation in the presence of the chromatin network, Zhang’s group has used a diploid human genome model parameterized with chromosome conformation capture (Hi-C) data. They have shown the important role played by the viscoelastic chromatin network to stabilize the multi-droplet state for nucleoli [Qi and Zhang (2021)].

Below we review a class of models that incorporate protein binding and have shown *de novo* predictive capabilities regarding 3D folding and dynamics of chromatin domains.

MiChroM model developed by Di Pierro et al. Di Pierro et al., 2016; Di Pierro et al., 2017; Di Pierro et al., 2018) captures protein binding induced phase separation implicitly by learning energy landscapes of chromatin folding from Hi-C and Chip-seq data once (Figure 2A). The potential energy function is trained by the application of a MaxEnt approach which generates the least-biased choice of parameters Lin et al. (2021). In addition to the usual potential of the homopolymer model, the energy function accounts for the interactions between chromatin types, the interactions between loop anchors, and a translational invariant compaction contribution Di Pierro et al. (2016). The combination of MaxEnt and machine learning has made the model stand out due to its simplicity and predictive power. There is now a dedicated server that, given the Chip-seq input, generates input files for molecular dynamics simulations Contessoto et al. (2020). Thanks to the transferable nature of the model, the subdiffusive dynamics of chromatin loci were naturally and qualitatively explained within this framework of epigenetically decorated chromatin A/B copolymer. The subdiffusive exponent originates from chromatin’s effective energy landscape shaped by thermodynamics-driven phase separation of A/B epigenetic types and non-equilibrium motorized cross-linking. Viscoelasticity and coherent motions of chromatin domains over multiple seconds were similarly seen as a consequence of the micro-phase separation of chromatin types.

The chromatin-binding proteins play an important role in the large-scale chromatin organization because they regulate the formation of compartments and mediate interactions between distal genomic regions *via* phase separation.

For instance, the Heterochromatin Protein-1 (HP1a), which is known for binding to epigenetically distinct nucleosomes, is now believed to play a crucial role in the heterochromatin formation Razin and Ulianov (2020); Llorens-Giralt et al. (2021); Eissenberg and Elgin (2014). Furthermore HP1 has been shown to undergo phase-separation both *in vitro* and *in vivo* Zenk et al. (2021); Larson et al. (2017). While the ability of HP1a in driving the formation of heterochromatin is clearly demonstrated for embryonic cells Zenk et al. (2021), for the differentiated cells HP1a appears to have different regulatory roles. We note that a detailed mechanistic picture of heterochromatin formation still remains full of puzzles calling for more detailed mechanistic modeling of chromatin Erdel (2020); Misteli (2020); Itoh et al. (2021b); Bhat et al. (2021). Besides HP1a the RNA molecules which bind to proteins and phase separate have also been found to have active involvement in the euchromatin domain formation through the microphase separation mechanism (Hilbert et al. (2021). In sum, there is now growing evidence that protein-induced microphase separation regulates the formation of chromatin compartments. Still, it remains unclear just how important are the various biophysical properties and interactions of phase separating proteins and RNAs in driving the formation of chromatin compartments. The strings and binders (SBS) model class of classic polymer models (Figure 2B) developed by the group of Nicodemi Nicodemi and Prisco (2009); Barbieri et al. (2012) was among the first to consider protein binding explicitly. In the SBS model, the chromatin is considered as a self-avoiding polymer that hosts several binding sites that interact with diffusing protein binders. The binding is controlled through a binding energy term which sets the overall binding energy scales and the affinities of binders. Specifically, within the SBS framework, a local microphase separation of cognate binding sites along 1D chromatin sequence was treated as the foundation for producing 3D architecture. This protein-induced microphase separation has been confirmed to be one of the major driving forces behind the formation of A/B compartments observed in Hi-C experiments. Since its inception, SBS has undergone a series of refinements which enabled making several remarkable predictions and explanations of chromatin folding specificity and variability in different organisms Bianco et al. (2020); Esposito et al. (2021). For instance, a tissue-specific *α*-globin genomic region was successfully recapitulated by the SBS model Chiariello et al. (2020). Recently, the variability of the TADs across single cells was explained in terms of the thermodynamic degeneracy of conformations predicted by polymer phase separation Conte et al. (2020).

Similar to Stringers and Binders, the Hip-Hop model proposed by Buckle et al. Buckle et al. (2018) combines explicit protein diffusion and binding with loop extrusion for predicting the 3D folding of genomic loci at a population and single-cell levels (Figure 2C). With a minimal *a priori* knowledge of epigenetic marks, the Hip-Hop model has been shown to recapitulate complex genomic loci in 3D and enable predictions of chromatin folding paths.

Living Chromatin model proposed by Haddad et al. (2017); Jost et al. (2017); Jost and Vaillant (2018); Tortora et al. (2020) considers epigenetic marks to the 3D folding of chromatin fiber (Figure 2D). Within this model, the formation of heterochromatin/euchromatin compartments in the *Drosophila* originates from the microphase separation of A/B types, which gives rise to a dynamical and stochastic organization chromatin. Interestingly, the model predicts weaker local self-interaction for euchromatin than for heterochromatin which favors more long-range and complex patterns of self-association. Authors calibrate the copolymer chromatin model from the MSD measurement to have a reliable description of chromosome folding kinetics. The living chromatin model is a combination of the copolymer chromatin and the epigenome regulation models Jost et al., 2012; Jost et al., 2017; Jost and Vaillant (2018). Each monomer can be in one of the three states: A, U, and I; the inter-conversion dynamics between these states result from random or recruited inter-conversions. The chain is modeled by a semi-flexible self-avoiding bead-spring model with specific short-range attractions between monomers of the same epigenomic states (A or I). Recruited conversions are achieved either by recruitment in cis 1D nearest neighbors or in the 3D neighborhood. There is also a noisy conversion between I and U and A and U and the possibility of external loading at some specific recruitment sites.

### 3 Mesoscale Models of Phase Separation and Dynamics of Chromatin Compartments

Its chain dynamics do not completely describe chromatin polymer because it is subject to a number of external physical constraints, mechanical and biochemical forces in the nucleus Aboelnour and Bonev (2021); Jerkovic and Cavalli (2021); Di Stefano et al. (2021b); Bhat et al. (2021). In particular, chromatin physically interacts with actin filaments, nuclear lamins, and ATP-powered transcriptional machinery such as RNA polymerase Zidovska, 2020a; Zidovska, 2020b. Recent studies have highlighted the important role played by actin and microtubule cytoskeletons in inducing nuclear membrane fluctuations and thereby facilitating chromatin mobility Almonacid et al. (2019). The role of ATP-powered polymerizes has also been shown to generate coherent motions and directed flows inside nuclei Saintillan et al. (2018). In the backdrop of all the internal and external forces acting on chromatin, the biomolecular phase separation appears as a ubiquitous mechanism for transmitting the forces whether directly or through biochemical feedback mechanisms Laghmach and Potoyan (2020). For instance, the transcriptional condensate formation and recruitment of RNA polymerase, formation of heterochromatin domains, and exertion of ATP-powered forces that locally restructure chromatin fiber are all processes that happen over scales where phase separation of chromatin domains is relevant and likely plays a major organizational role.

How to describe and disentangle the motions of chromatin in the cell nucleus that result from the collective action of forces spanning vastly different scales and operate in a highly heterogeneous and out of equilibrium environment of the nucleus? This is certainly a very challenging problem and is most likely to develop several innovative approaches that could combine innovative computational models and integrate them with data from imaging experiments. A key challenge in modeling chromatin dynamics at the scale of the full nucleus is finding the appropriate resolution for capturing processes taking place over spatial and temporal scales of interest.

A promising approach is to employ field-theoretical and continuum-based models that coarse grain over chromatin’s particular nature and resolve it as a viscoelastic complex fluid mixture occupying the nucleus. Some of the earliest field-theoretical approaches for studying chromatin dynamics were provided by Bruinsma et al. Bruinsma et al. (2014). In this study, a theoretical framework based on the linear response theory was applied to a binary viscoelastic fluid to analyze studies of ATP-driven chromatin dynamics. A toy model demonstrated the relationship between chromatin density and velocity correlation with the viscoelastic moduli of the chromatin solution.

Recently, phase-field models have been finding increased application for studying cell biology ranging from mechanics and motility of individual cells to multicellular systems Nonomura (2012); Najem and Grant (2016); Akiyama et al. (2018); Jiang et al. (2019); Moure and Gomez (2021). The first phase-field model for multicellular systems was proposed by Nonomura Nonomura (2012). Modeling nuclear interior and dynamics of chromatin compartments is another natural application for phase-field models since the problem can be described in various moving chromatin interfaces that are segregated from one another. The chromatin state in the phase-field models can be resolved with an arbitrary number of chromatin types corresponding to heterochromatin/euchromatin or facultative/constitutive heterochromatin forms. The primary driving forces for emergent nuclear architecture and dynamics are derived from the microphase separation of heterochromatin sub-types, the surface tension of chromatin droplets, and differential affinity for chromatin-lamina interactions. Voltage and surface constraints are imposed on chromatin types to capture chromosomal and nuclear boundaries. Given the dense, active, and heterogeneous nature of nuclear chromatin, it is worth highlighting the advantage of field-theoretic description, which manages to avoid the notorious glassy states encountered in the particle-based polymer simulations, thereby facilitating the study of long-timescale chromatin dynamics and patterning at the scale of whole nucleus Shi et al. (2018); Kang et al. (2015).

**FIGURE 3:**
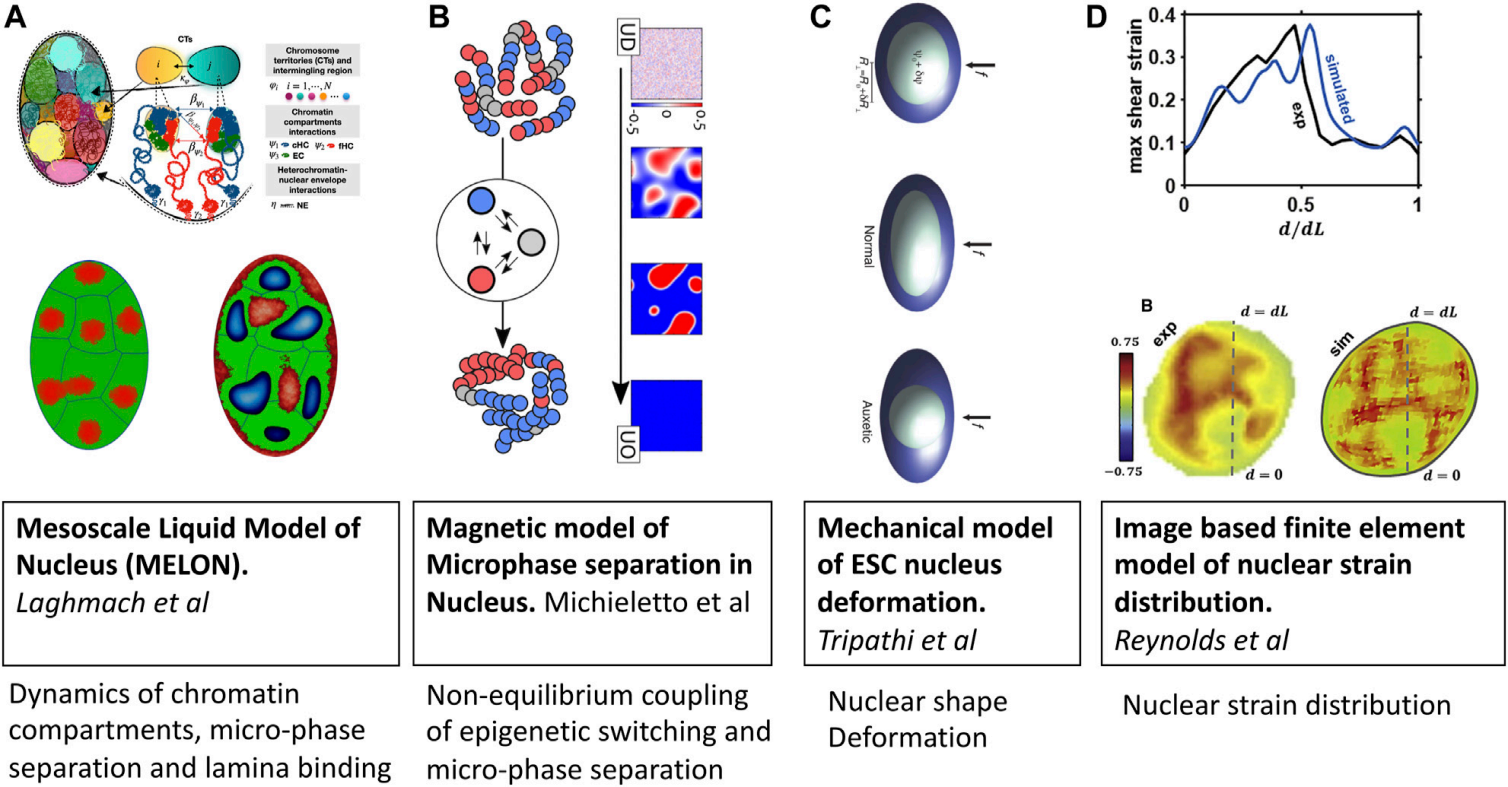
Mesoscale models of eukaryotic nucleus. Images are adopted from original papers with copyright agreement. From left to right; **(A)** Mesoscale liquid model of nucleus Laghmach et al. (2020), Laghmach et al., 2021, **(B)** Magnetic model of chromatin phase separation by Michieletto et al (2019), **(C)** Mechanical model of stem cell nucleus deformation Tripathi and Menon (2019), and **(D)** Image based finite element model of nucleus Reynolds et al. (2021).

Mesoscale liquid model of nucleus (MELON) developed by Laghmach et al. Laghmach et al. (2020); Laghmach et al., 2021) describes the state of the nucleus as a mixture of incompressible multiphase fluids (Figure 3A). In this description, the emergent chromatin patterns are seen as an interplay of phase separation, chromosomes’ territorial affinity, and surface tension of heterochromatin-euchromatin droplets. The significance of the surface tension of heterochromatin within the nucleus and its interaction with heterochromatin determines the nuclear morphology reminiscent of senescent, inverted, and conventional architectures. The model also has provided insights into how remodeling nuclear volume has dynamical implications for chromatin compartments where the kinetic barrier for phase separation is lowered thanks to the proximity of heterochromatic centers in adjacent chromosomal territories. This mesoscale perspective offers new avenues for integrating imaging experiments and reconciling dynamic phenomena observed in *in-situ* nucleus imaging. Using the MELON model with a ternary liquid-like chromatin state has looked at dynamics of chromatin compartment during interphase. Addressed dynamics of chromatin compartment formed by phase separation of constitutive and facultative heterochromatin, which lead to gel-like elongated channels and spheroidal droplets centered chromosome territory Laghmach et al. (2021).

Similar to the design of the living chromatin model discussed in the previous section, the group of D. Marenduzzo Michieletto et al., 2016; Michieletto et al., 2019; Coli et al. (2019) has also developed a series of models with explicit coupling between 1D epigenetic dynamics and 3D folding of chromatin (Figure 3B). The key difference from other explicit protein models is that epigenetic changes are modeled *via* spin-Hamiltonian dynamics instead of biochemical reactions. The application of magnetic models of chromatin has shown the importance of non-equilibrium processes in regulating chromatin domain patterning, which arrest microphase separation of euchromatin-heterochromatin in lifelike morphologies instead of falling into equilibrium dominated by either of chromatin types Michieletto et al. (2019).

An emerging area where mesoscale models of chromatin will be instrumental is cell mechanotransduction and mechanosensing. To understand the relationship between local DNA density and mechanical stress fields generated in the nucleus, Reynolds et al.Reynolds et al. (2021) have set up a mesoscale three-dimensional finite-element model of a cell nucleus from image stacks collected by confocal microscopy (Figure 3C). Simulations suggest that the mechanical behavior of the nucleus is highly heterogeneous, with a non-linear relationship between local chromatin packing and shear moduli. They also find that disruption of the nuclear envelope associated with lamin A/C depletion significantly increases nuclear strain in regions of low DNA concentration.

The coupling between chromatin compaction states and nuclear shape has been studied by Tripathy and Menon Tripathi and Menon (2019) using a mesoscopic mechanical model of the nucleus that resolves nuclear shape variables and applied force accessing in AFM experiments (Figure 3D). Armed with this mesoscale mechanical model of the nucleus, the authors explain the measurements of the deformability of cell nuclei in the transition state between embryonic stem-cell state and the differentiated state of mouse stem cells which have a negative Poisson’s ratio. A key insight from the study is that fluctuations in chromatin compaction are coupled to fluctuations in the relatively soft nucleus’s dimensions that characterize stem cells.

Another notable mesoscopic mechanical model of the nucleus has been developed by Marko and colleagues Banigan et al. (2017); Stephens et al. (2019) to understand force-extension regimes when stretching the nucleus and imaging the lamina of isolated cell nuclei. The model can explain the two linear tension-strain regimes, corresponding to a weak, linear elastic response to small tensions and a stiff linear response to nuclear deformation, which deforms sufficiently to align the inter-subunit bonds with the tension axis. Also, the model predicts the buckling transition between the two regimes.

In conclusion, we would also like to mention mesoscopic models that go beyond a lifetime of a single nucleus in order to probe the dynamics of the establishment of heterochromatin compartments and mechanisms of propagation of “epigenetic memory” through cell divisions Ng et al. (2018); Feinberg and Irizarry (2010); Himeoka and Kaneko (2020); Nickels et al. (2021). Mathematical models formulated as kinetic monte carlo Dodd et al. (2007); Nguyen et al. (2021) or agent-based Sneppen and Ringrose (2019) dynamical simulations have been widely used to study the stochastic dynamical interplay of local and global feedback mechanisms of histone methylation and acetylation along the 1D genomic sequence. While often lacking in spatially resolved details compared to polymeric and phase-field models, these stochastic kinetics studies have nevertheless offered the first glimpse into systems-level regulation of heterochromatin formation, maintenance, and regulation.

A recent study employing experiment and mathematical modeling Nickels et al. (2021) has looked at the fission yeast cell, which contains nucleation center of heterochromatin CenH. By incrementally varying the meting region size, the mathematical model aided by the experiments has come across a remarkable finding that heterochromatin propagation occurs in an all-or-none fashion, where the entire domains collapse abruptly. This stochastic burst-like mode of heterochromatin domain fashion goes against the linear propagation mechanism and necessitates the inclusion of distance-dependent kinetic processes. Future studies would be imperative to include spatially resolved and global feedback mechanisms that act on histone modifications. Since the mathematical models of epigenetic processes are rather extensive, we refer the readers to excellent books and reviewers which cover the accumulated knowledge and latest insights from mathematical modeling of emergent trans-generational epigenetic memory and establishment of chromatin domains from first principles stochastic kinetics of histone marking and erasure Sneppen (2014); Ringrose (2017); Menon et al. (2021); Lövkvist and Howard (2021).

## 4 Conclusion

Given the complex, crowded, and out of equilibrium nature of the nuclear environment, it is challenging to distill the driving forces of chromatin patterning and its dynamical evolution. High-resolution imaging studies have shown a much more stochastic, heterogeneous, and dynamical chromatin nature than expected. At the same time, ideas revolving around protein and RNA-induced phase separation have given us with mechanistic clues to rationalize the emergent nuclear architectures, and dynamical observables probed in imaging experiments. In this respect, mechanistic models incorporating physical intuition and empirical data prove crucial for interpreting and guiding future experiments.

Going forward we see a number of conceptual and methodological bottlenecks overcoming of which will take our understanding of chromatin organization and dynamics to the next level. (I) On the conceptual side, it is becoming evident that there are different chromatin compartments that have heterogeneous properties and different mechanistic paths of formation. Therefore grouping them under one umbrella and attempting to explain behavior through the lens of phase separation alone is not realistic. Hence, more systematic differentiation and classification schemes need to be developed in part by imaging different cell lines and updating the vocabulary of theoretical models of intracellular phase separation. (II) On the methodological side, as polymer models of chromosome 3D folding are becoming more established and predictive thanks to Hi-C experiments, we need to break new ground in the direction of mesoscale modeling techniques for characterizing chromatin patterning dynamics seen in high-resolution imaging experiments.

In summary, promising future directions are emerging where an integrative approach combining novel experimental biophysical imaging methods of nuclear chromatin, mechanistic mesoscale models together with the development of machine learning and analytic data methods will be instrumental in the quest for a deeper understanding of the nature of chromatin patterning and its functional implications.
